# A Review of Technology-based Interventions in Improving Type 2 Diabetes Management in Chinese Americans

**DOI:** 10.31372/20190401.1018

**Published:** 2019

**Authors:** Wen-Wen Li, Jenny Zhong

**Affiliations:** a San Francisco State University, San Francisco, CA 94132, USA

**Keywords:** racial ethnic minorities, Diabetes Mellitus Type 2, Chinese American, management, telemedicine, telemonitoring, telehealth, e-healthcare, telehealthcare, HbA1c

## Abstract

Health disparities of type 2 diabetes (DM2) in America is an ongoing crisis. Despite this, technology has been helpful in managing DM2 in the non-Hispanic White, Hispanic, and African American populations and has been proven effective. Furthermore, it may be used to supplement health provider DM2 care through telemedicine to lower hemoglobin A1c (A1c), a gold standard DM2 measurement, and other DM2-related outcomes, such as glycated hemoglobin. The purpose of this study was to review current literature on the use of telemedicine in assisting DM2 management in racial ethnic minorities and to discuss how to adjust the telemedicine DM2 management program to be applied to Chinese Americans. In addition, it is worthy to note that the role of nurses makes a substantial difference in the effectiveness of technological management of DM2 from being culturally sensitive and sending catered messages to address specific patient needs.

## Introduction

Health disparities among racial ethnic patients with type 2 diabetes (DM2) is a widespread concern in the United States (Centers for Disease Control and Prevention, 2017; Chow, Foster, Gonzalez, & McIver, 2012). According to the Centers for Disease Prevention and Control (CDC), 30.3 million people have diabetes (Centers for Disease Control and Prevention, 2017). The Asian immigrant population in particular has had a steady increase in DM2 diagnoses within the last several decades, second only after Mexican immigrants, who had the highest increase. (Pew Research Center, 2015). Specifically, the Chinese immigrant population has had a six-fold increase since 1980, reaching 2.3 million people in 2016 (Zong & Batalova, 2017). As the numbers increase, so too do the amount of Chinese diagnosed with DM2, which adds to the ongoing health disparity.

Chinese Americans, who are foreign-born Chinese or of Chinese descent, are the largest Asian ethnic group in the United States but suffer up to twice the rate of DM2 compared to European Americans (Joslin Diabetes Center, 2016; Zong & Batalova, 2017). Even with a lower body weight, Asian Americans are more likely than Caucasians to have DM2 with screening recommendations of a body mass index (BMI) of 23 for Asian Americans compared to 25 for Caucasians due to their higher risk (Abid, Ahmad, & Waheed, 2016). 

The prevalence of DM2 in Chinese Americans is significantly higher at 5.4% when compared to non-Hispanic Whites at 2.9% (Gupta, Wu, Young, & Perlman, 2011). A major contributing factor may be due to a difference in body fat distribution where Asians have a higher propensity to develop visceral versus peripheral adiposity (Hsu, Araneta, Kanaya, Chiang, & Fujimoto, 2015). In other words, Asians—including Chinese—have a higher chance of developing DM2 at a lower BMI when compared to non-Hispanic White.

The ongoing financial burden on the health care system due to diabetes alone has resulted in a total cost of $327 billion in 2017 (American Diabetes Association, 2018). Patients with diabetes account for 30% of all inpatient hospital days, 13% of physician visits, and 30% of prescription medications to treat complications (e.g., heart attack and stroke) (American Diabetes Association, 2018). Despite these discouraging numbers, there may be a solution to address the ever-increasing diabetes epidemic. Innovative technology, such as the use of internet and mobile devices for DM2 management, is quickly becoming a noticeable trend because of positive outcomes. One of those outcomes is the added convenience of being able to access care beyond clinics or medical offices, using delivery of disease-related services and information through telephone, internet, computer, and/or mobile technology. These telemedicine technologies support long-distance clinical health care, patients, and health professionals (Fitzner & Moss, 2013).

Currently, there is limited research related to telemedicine or e-healthcare targeting DM2 in racial ethnic minorities, such as Chinese Americans. Telehealth DM2 research on minorities has only been done with mostly African Americans and some Hispanics, albeit with efficacy (Carter, Nunlee-Bland, & Callender, 2011; Davis et al., 2010). With the higher risk for DM2 in Chinese Americans, the use of technology to supplement health care is a promising solution to care for those already diagnosed with DM2 (Joslin Diabetes Center, 2016). DM2 telehealth interventions have been shown to successfully complement traditional one-on-one patient care. They significantly improved blood sugar control by as much as a 0.5% hemoglobin A1c (HbA1c), a gold standard DM2 measurement, decrease over six months as noted in a multinational population with DM2 (Welch, Balder, & Zagarins, 2015).

Examples for using telemedicine to help Chinese Americans with DM2, especially those who live in a rural area or in smaller communities, such as Chinatown, include remote monitoring of vital signs, the use of interactive videos, and consumer-focused wireless applications. Telemonitoring has been shown to improve healthcare access, decrease costs, and enhance quality of care (Pare, Jaana, & Sicotte, 2007. Other combined interventions with telemedicine may also include follow-up telephone calls or other forms of individualized communication with a nurse (e.g., culturally and linguistically appropriate) to further provide education, support, and collaboration with the patient (Pare et al., 2007). These factors may enable Chinese Americans to monitor and stay connected to their own health while decreasing burdensome and costly visits to their primary healthcare provider.

Researchers and health care practitioners are finding ways to incorporate the use of technology to improve healthy eating, monitoring, and taking medicine along with other DM2 self-management tools in non-Hispanic White US populations (Fitzner & Moss, 2013). It has been shown that the use of technology can effectively and efficiently offer affordable ways to support minorities with DM2 (Fitzner & Moss, 2013). Because little research was found on Chinese Americans, the purpose of this systematic review was to examine the efficacy of technology-based interventions in managing DM2 in racial minority populations. Since the majority of published studies focus on African American and Hispanic American minority populations, this systematic review was an opportunity to glean interventions that worked for these populations. But further evaluation was done to help determine if some of the interventions are feasible and applicable to the care of Chinese Americans with DM2.

## Methods

**Figure 1:**
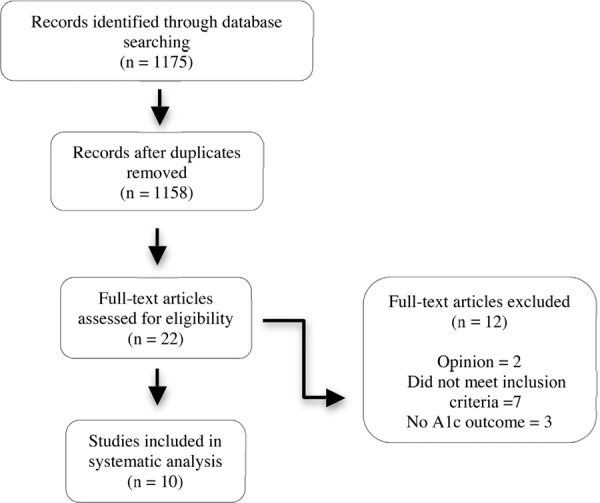
Flowchart depicting literature selection.

This comprehensive systematic review was based on the guideline developed by the research team, depicted in Figure 1. From fall 2017 to spring 2018, PubMed, CINAHL, ScienceDirect, ProQuest, Google Scholar, and the Cochrane Central Register of Controlled Trials databases were searched for clinical trials that were published from 2010 to 2018 and studied technology-based interventions for DM2 management in racial ethnic minorities. The following search terms were used alone and in combination: “racial ethnic minorities,” “immigrant,” “type 2 diabetes,” “DM2,” “black,” “Hispanic,” “Chinese,” “American,” “management,” “telemedicine,” “telemonitoring,” “telehealth,” “randomized clinical trial,” “randomized controlled trial,” “e-health,” “m-health,” “telehealthcare,” and “HbA1c,” among others. The developed guideline was used to identify and evaluate eligible studies. A manual search was also done on the reference lists of several relevant studies for review. Duplicate studies were not included. The question, “How can we incorporate the use of technology for Chinese Americans to effectively manage their DM2?” provided the focus for the literature search.

Inclusion criteria for this review included: (1) original research study with telemonitoring of HbA1c or glycated hemoglobin levels of patients with DM2; (2) study which meets quality appraisal criteria (please see the next section for details); (3) inclusion of an outcome measure of HbA1c, oral glucose tolerance test (OGTT), or fasting plasma glucose levels; (4) published studies in peer-reviewed journals; (5) studies from all countries that were written in English; (6) studies with a quantitative experimental design involving participants with DM2, and; (7) participants over 18 years of age.

Two independent researchers reviewed the abstracts. The studies regarding telemanagement of DM2 were thoroughly assessed under the developed guideline. A total of 1,175 articles were retrieved after the initial search; these were screened by title and removed if keywords regarding technological interventions for DM2 management were not present. More articles were excluded if they did not meet the inclusion criteria. Editorials, commentaries, anecdotes, and literature reviews were also excluded. As a result, 10 articles which met the criteria were used for final analysis and discussion. These 10 articles addressed the topic of telemedicine to improve care of DM2 in racial ethnic minorities, which was the focus of our review (Figure 1).

### Systematic Review

A total of 10 articles were included in this review (Table 1). All 10 articles were studies targeting an ethnic community (a majority were African Americans, Hispanic, and Native American) with DM2. Table 1 shows the comparison of studies using racial ethnic populations in this systematic review.

**Table 1:** Description of Studies Included in Systematic Review

Study/design	Recruitment rate/incentive	Type/intervention description	Purpose/use of nurse in study?	Sample size/ethnicities/duration	Geographic region/setting	Specific outcomes for racial/ethnic groups/brief results
Arora et al. (2014) Open-label RCT	**Recruitment rate:** 72% **Incentive:** $175/per subject	**Intervention:** Unidirectional text message sent by research staff Type of intervention: Mobile, TExT-MED: Daily Text message triggers for patient motivation and self-care **Control:** Usual care	**Purpose:** To determine if a unidirectional, text message is effective in improving clinical outcomes like A1c and decreases in emergency department use **Use of nurse:** no nurse, research assistants only	*N* = 128 **Gender:** Female 64% **Ethnicities:** Hispanic/Latino 87%; Black 9%; Asian/Pacific Islander 2% **Duration:** 6 months	University Hospital Emergency Department in Los Angeles, Southern California, USA	**Major outcome:** A1c decreased by 1.05% in intervention group compared with 0.60% in control **Secondary outcomes:** Medication adherence and A1c changes were stronger among Spanish speakers, decrease in ED use. However, the intervention did not result in statistically significant improvement in A1c
Capozza et al. (2015) RCT	**Recruitment rate:** 78% at 90 days, 62% at 180 days **Incentive:** none mentioned	**Intervention:** Bidirectional text message, sent by research staff Type of intervention: Mobile; seven DM2-related text messages/day developed based on American Diabetes Association clinical practice guidelines **Control:** Usual care	**Purpose:** To test the impact on glycemic control of a two-way text messaging program is effective in improving A1c **Use of nurse:** no nurse, research staff only	*N* = 93 **Gender:** Female 61.3% **Ethnicities:** 70% White; 3% Hispanic; 2% Black; 3% Asian; 1% Pacific Islander; 20% other **Duration:** 6 months	Primary care clinics in Salt Lake City, Utah, USA	**Major outcome:** No significant A1c difference between control and intervention group (*P* > 0.05) **Secondary outcomes:** Patients reported high satisfaction with the intervention program all scoring 3 or above (on a 4-point scale)
Chung et al. (2017) Retrospective analysis study	**Recruitment rate:** 100% **Incentive:** none	**Intervention:** Internet **Control:** none	**Purpose:** To determine if messaging is associated with use and A1c among patients with DM2 **Use of nurse:** no nurse, providers only	*N* = 37,762 **Gender:** Female: 43% **Ethnicity:** 40% White, 3% Black, 32% Asian, 5% Latino, 21% other **Duration:** 3-year span (2011-2014)	Health records of mixed payer, multispecialty outpatient (Stanford Hospital) practice in Northern California, USA	**Major outcome:** Secure message improved DM2 outcome (i.e., A1c; eye health). In addition, message users are more self-motivated and was associated with better A1c. Non-message users had less change in A1c
Davis et al. (2010) RCT	**Recruitment rate:** 81% **Incentive:** gift card- unknown amount	**Intervention:** Remote comprehensive DM2 self-management education intervention, DM2 TeleCare: videoconference, telephone, fax, retinal camera Type of intervention: in-person and videoconference with self-management team **Control:** 20-minute DM2 session by nurse	**Purpose:** To determine if a remote comprehensive, telemedical DM2 self-management education (DSME) is effective in improving A1c **Use of nurse:** yes, also dietitian and certified DM2 educator	*N* = 165 **Gender:** Female 74.6% **Ethnicities:** 75% African American, 24% White **Duration:** 1 year	Rural South Carolina-federally qualified health center, USA	**Main outcome:** A1c results were significantly reduced from 9.4%. At 6-months −1.1%, at 12-month −1.2%, while control group had a reduction of −0.2% only at 6 and no change at 12 months
Glasgow et al. (2012) RCT	**Recruitment rate:** 81.8% usual care, 68.6% computer-assisted DM2 self-management (CASM), 74.7% CASM plus human support (CASM+) **Incentive:** none mentioned	**Intervention:** Internet Type of intervention: Online website or Online website + interventionist enhanced social support (follow-up calls and group meetings) **Control:** Usual care with computer-based health risk appraisal feedback and recommended behaviors	**Purpose:** To determine if Internet-based, CASM intervention compared to a CASM+ condition helps reduce A1c **Use of nurse:** no, only research staff coordinator	*N* = 463 **Gender:** Female 49.8% Ethnicity: 21.8% Latino, 72% White, 15.4% Black, 1.6% Asian; Native American 6.7% **Duration:** 12 months	Colorado, 5 primary care clinics within Kaiser Permanente, USA	**Main outcome:** At 12 months, the intervention group average A1c decreased 0.02% while control group decreased 0.12%; outcomes were non-significant
Greenwood et al. (2015) RCT	**Recruitment rate:** 100% **Incentive:** $10 gift card; glucometer, test supplies, USB cables	**Intervention:** Mobile & Internet Intervention touchscreen tablet + glucometer via USB cables for 84 daily health questions; health sessions through Care Innovations guide **Control:** Booklets, telephone/secure messaging contact with provider	**Purpose:** To determine if a telehealth remote monitoring intervention using paired glucose testing and asynchronous data analysis in adults with DM2 reduces A1c **Use of nurse:** nurse care coordinator	*N* = 90 **Gender:** 46.7% females Ethnicities: 64% White, 15.7 Hispanic, 0.3% Black, 20% other **Duration:** 6 months	California large health care system	**Main outcome:** Significant A1c decrease of −1.11% from 8.5% in the intervention group compared to −0.70% in control group from 8.2% at 6 months
Quinn et al. (2016) Cluster RCT	**Recruitment rate:** 100% **Incentive:** glucometer & $250/provider/patient	**Intervention:** Mobile phone app Type of intervention: Patient-coaching system: software application and web portal; educational/behavior/motivational messaging responding to patient entered data **Control:** usual care	**Purpose:** To determine if mobile coaching system has effects on A1c levels in younger (<55) vs older (^3^55) DM2 patients **Use of nurse:** none; through direct provider contact or virtual DM2 educators	*N* = 163 **Gender:** 50.3% females **Ethnicities:** White 52.7%, Black 39.3%; other 8% **Duration:** 1 year	Maryland, USA: Four primary care practices	**Main outcome:** There was no significant difference between either age group, both had a decrease in A1c at the end of the (one year) study with −2.0% of baseline 9.9% for <55 years old and −1.7% of baseline 9.8% for group >55 years
Tang et al. (2013) RCT	**Recruitment rate:** 87% **Incentive:** glucometer - Palm Treo smartphone	**Intervention:** Motivational wireless glucose reading feedback, online messaging with health team **Type of intervention:** Wireless glucometer capable of uploading: patient records; online care team messaging and support; text message and video education **Control:** usual care	**Purpose:** To determine if online disease management system supporting patients w/uncontrolled DM2 reduces A1c **Use of nurse:** nurse care manager	*N* = 450 **Gender:** 40% female **Ethnicity:** Hispanic 9.4%, Race: White 58.5%; Black 5%; Asian 21.4% **Duration:** 12 months	Palo Alto, CA, USA. Palo Alto Medical Foundation nonprofit	**Main outcome:** A significant decrease at 6 months with A1c of −1.32% at baseline 9.28% in the intervention group compared to control of −0.66% at baseline 9.24%. Not significant at 12 months with intervention of −1.14% compared to control of −0.95%
Vuong et al. (2012) Ongoing case study	**Recruitment rate:** 96% **Incentive:** PDA to use	**Intervention:** Personal digital assistant (PDA) Type of intervention: Use of PDA for DM2 self-management e-interventions **Control:** Usual care	**Purpose:** To determine impact of personal digital assistant (PDA) features, acceptability, and usability in e-health DM2 self-management **Use of nurse:** none; project coordinator only	*N* = 376 **Gender:** 60% females **Ethnicities:** 50% minority (25% African Americans, 25% Hispanics) **Duration:** 6 months	Ongoing study of at least three years: Texas, USA	**Main outcome:** A1c results were not reported. However, PDAs reduced A1c for those who were tech savvy but in general had low usability and perceived ease of use due to being not straightforward and non-user-friendly
Welch et al. (2015) Single sample, pre-post study	**Recruitment rate:** 96.6% **Incentive:** glucometer, BP cuff, electronic pillbox	**Intervention:** Remote home monitoring (RHM) with electronic pillbox and blood pressure monitor through bluetooth **Type of intervention:** RHM use with minimal nurse outreach (3 brief calls) **Control:** none	**Purpose:** To determine A1c reduction of a 3-month telehealth program for poorly controlled DM2 patients **Use of nurse:** yes	*N* = 30 **Gender:** 56.7% females **Ethnicities:** 73% African American, 26% Latino **Duration:** 3 months	Urban low-income community health center located in Connecticut	**Main outcome:** Significant A1c improvement of 0.6% from a baseline level of 8.3% (*P* < 0.05) at 3 months

### Methodological Quality

**Study design**. Our search effort was focused on obtaining information from a Randomized Controlled Trial (RCT) as it is considered as the highest level of evidence (Moher, Liberati, Tetzlaff, Altman, & The PRISMA Group, 2009). Based on this attempt, the articles included seven RCTs, one retrospective analysis study, one case study, and one single sample, pre and post intervention study (see Table 1). Thus, our systematic review was primarily based on RCTs (7 out of 10 studies).

**Study duration**. Studies ranged between 3 months to 3 years with an average study length of one year. A majority (80%) of the studies to test feasibility and effectiveness took between 6 and 12 months. A 6 to 12 month observation period is necessary because DM2 requires continuous, long-term management to see significant results [e.g., the mean difference in HbA1c dropped 0.40% (95% CI = −0.65, −0.15) from baseline to 6-month follow-up using culturally competent health coaches to help patients manage their DM2 (Ivey et al., 2012)].

**Risk of bias**. Some studies were at risk for bias due to their lack of blinding of the study participants and missing data which may have consequently affected the validity of results. For the RCTs used in this analysis, randomization was either done using computer-generated randomization, or were individually randomized via a computer program developed by a computer programmer and statistician as in the study by Glasgow et al. (2012). The study by Quinn et al. (2016) used cluster randomization (such that an entire physician practice site was randomized to the same study group) to prevent potential contamination of the study interventions.

Incomplete data may also account for bias, and this usually occurred due to attrition of participants from loss to follow-up, dropping out, or failing to complete background information. To account for missing data, Glasgow et al. (2010) included the use of *a priori* comparisons and use of generalized estimating equations (GEE) models (Glasgow et al., 2010). These models were specifically for longitudinal data—and imputation analyses, which can estimate missing data to more accurately represent the target population. Additionally, social desirability bias may have been present in the study by Arora, Peters, Burner, Lam, and Menchine (2014) due to subjects replying favorably to the investigators because they think the investigators want them to.

**Sample size**. The sample sizes of the studies ranged from 30 to 37,762 participants, however, the majority of studies had a decrease in the number of participants due to a lack of follow-up. Arora et al. (2014) had a 28% loss to follow-up, but accounted for this loss by increasing the number of participants recruited. In a study by Davis et al. (2010), the original intended sample size was 200, based on a power of 0.8, *α* of 0.05, and an effect size of 0.5% change in HbA1c as the primary outcomes. The detectable clinically relevant changes in secondary outcomes and allowing conservatively for 30% loss to follow-up, yield a final sample size of 165. Tang et al. (2013) had their study design powered to detect net HbA1c improvements of 0.5% or greater. Hence, a sample of 200 per arm was calculated to provide 91% power to detect an effect size of 0.3, assuming a 15% loss in 12-month follow up (Tang et al., 2013). The sample size resulted in 415 patients, which was more than their initial goal. All the aforementioned studies showed adequate power to determine the efficacy of the intervention, except the study by Welch et al. (2015). In this study, Welch et al. (2015) utilized a single group, pre-post intervention design with a sample size of 30. Although the sample size was small, they rigorously designed their study through targeting clinics with a diverse population of ethnic and socioeconomic groups with very high [96.6%] completion rate. The results showed their intervention was efficient in reducing HbA1c which improved 0.6% from a baseline level of 8.3% (*P* < 0.05).

**Inclusion criteria and recruitment**. Inclusion criteria for each study consisted of subjects with one year of DM2 diagnosis, HbA1c between 7% and 11% in the past 6 months, age 18 or older, and no serious psychiatric or mental health complications present. Participants were English speakers, had internet or email access, had electronic devices with texting function, were not pregnant, and were able to provide informed consent. Recruitment was primarily through primary care clinics. As for the recruitment strategies, Glasgow et al. (2010, 2012) sent physician letters to prospective participants and oral referrals by physicians and patient educators (Glasgow et al., 2012; Glasgow et al., 2010). A recruitment rate of 37% was considered efficient (Glasgow et al., 2010). According to Capozza et al. (2015), recruiting patients to enroll was difficult primarily because patients were unfamiliar with texting, and those with lower technology proficiency typically required 15–30 min of one-on-one support to fully understand the enrollment process for the study. Welch et al. (2015) targeted poorly controlled DM2 patients seen at a local community health center but did not specify what method was used to recruit participants.

**Inclusion of ethnic minorities**. All studies included participants who were of ethnic minority backgrounds, and half of them (five out of ten studies) had a sample population of more than 50% of racial minority descent. The top three racially diverse studies were by: Arora et al. (2014), with 87% Hispanic/Latino, 9% Black, 2% Asian/Pacific Islander; Davis et al. (2010), with 75.3% African American/other; and Welch et al. (2015), with 73.3% Black/African American, 26.7% Hispanic ethnicity, 3.3% other. Though none of the studies specified inclusion of Chinese Americans, the two studies with the most Asian participants were Chung, Panattoni, Chi, and Palaniappan (2017) and Tang et al. (2013) with 32% and 21%, respectively. However, neither study broke down the specific types of Asians included in the study.

**Incentives**. A majority (70%) of the studies included incentives for the participants. In general, the studies which provided incentives had a better recruitment rate, ranging from 72% to 100%. The studies which did not provide incentives were the retrospective projects. There were multiple items used as incentives including: (1) gift cards or checks; (2) a glucometer and glucose testing supplies and USB cables; (3) any of monitoring devices, such as a blood glucose (BG) monitor, an automatic blood pressure (BP) cuff, or a MedMinder” pillbox—a user-friendly cellular pillbox marketed by Ideal Life^®^ (Toronto, ON, Canada). In terms of gift cards or checks, Greenwood, Blozis, Young, Nesbitt, and Quinn (2015) gave each study participant $10 at the end of the study with the recruitment rate of 100%. Quinn et al. (2016) reimbursed providers modestly ($250/patient enrolled) for their research efforts, and the recruitment rate was 100%. Arora et al. (2014) compensated all subjects regardless of treatment allocation with $175 for time and travel costs associated with study follow-up visits, and some subjects were given $20 per month to upgrade their phone carrier plans to unlimited messaging. Arora et al.’s (2014) recruitment rate was 72%. Welch et al. (2015) gave each participant a BG monitor, an automatic BP cuff, and an electronic pillbox, yielding a recruitment rate of 97%.

### Telemedicine Interventions, Measures, and Outcomes

**Telemedicine intervention**. Across all groups, the primary goal was to achieve BG control by using telemonitoring to communicate BG data. All studies utilized telemonitoring, through various methods, including text messaging, video conference, and self-reporting of BG by participants using a BG device, such as a glucometer, or through electronic health databases in addition to standard medical care. Another method involved obtaining BG readings and other standardized data—like blood pressure, insulin dose and weight—by nurses, research assistants or physicians at specific intervals as dictated by the study protocol with a remote home monitoring program through use of glucometers connected wirelessly with a Bluetooth adaptor, for example, to transmit data to online databases.

The retrospective study by Chung et al. (2017) analyzed secure patient-provider messaging through a patient portal to determine if physician advice is associated with fewer face-to-face visits and better DM2 management. Outcomes of that study concluded that secure messaging in addition to face-to-face visits were associated with better DM2 clinical processes and outcomes.

Davis et al. (2010) randomized participants to either a 13-session DM2 Telecare intervention or to usual care. DM2 Telecare involved the use of a dietitian and nurse/certified diabetes educator (DM2 education team) for remote comprehensive DM2 self-management education. Of the 13 sessions, three were conducted in-person while the rest were conducted by interactive videoconferencing, either individually or by group. Participants completed daily logs recording the results of self-monitored BG, diet, and physical activity with a pedometer. Additionally, participants were offered optional retinal imaging and were referred to an ophthalmologist for any abnormal findings. Control group participants received one 20-min DM2 education session using ADA materials, conducted individually at the time of randomization by a nurse. Primary outcomes (HbA1c) and secondary outcomes (LDL cholesterol and the albumin-to-creatinine ratio) were collected on all randomized participants at baseline, at 6, and at 12 months.

In the study by Tang et al. (2013), intervention group participants were provided with wireless remote monitoring tools of BG and/or BP readings, where data were uploaded to an electronic health record and analyzed by the personalized healthcare program system. Participants communicated online with healthcare team members who could provide individualized, culturally sensitive advice, make protocol-based changes to medications, and send patient-specific text messages and video educational clips. Clinical measurements and online questionnaires were collected from all participants at baseline and also at 6 and 12 months. Participants were also provided with wireless remote monitoring tools and enhanced patient portal functions. Arora et al. (2014) randomized the emergency department (ED) patients to the text message-based mobile health intervention (TExT-MED; the intervention group) in which two daily unidirectional text messages in English or Spanish were distributed regarding DM2 management for 6 months in addition to their usual care. Examples of unidirectional text messages included: “Having diabetes can lead to a heart attack or stroke—but it doesn’t have to” or “Medication reminder! Don’t leave home without your medications” (Arora et al., 2014). Outcome data, including HbA1c level, self-reported medication adherence, self-efficacy, performance of self-care tasks, quality of life, and DM2-specific knowledge, were collected at enrollment and at 6-month follow up. Conversely, Capozza et al. (2015) had a two-way text messaging program for the intervention group (Care4Life) which received from one to seven DM2-related text messages per day depending on the choices they made at enrollment in addition to their usual care. Here, participants could respond to the texts with a “yes,” “no,” or numerical answers which would trigger another chain of responses. Participant’s biometric and behavioral information were stored in a Web-based portal where study participants could view trends and print out reports to bring to clinical care visits. Primary outcome data of HbA1c were collected at baseline, 90 days, and 180 days while secondary outcome data of patient interaction and satisfaction with the program was assessed at 90 and 180 days.

In Greenwood et al. (2015), the study was a two-group RCT with 1:1 randomization to the telehealth remote monitoring with paired glucose testing or control group. The intervention group attended a one-hour, small group, in-person training session led by the certified diabetes educator where they were trained on the use of a joint touchscreen computer-tablet glucometer, implementation of the complete feedback loop, and use of paired glucose testing, in addition to other self-monitoring guidance. They were also asked to check blood glucose before and two hours after the same meal, to do physical activity for one week, and to create a behavior change action plan. Data entered in the tablet were downloaded as needed via Internet by certified diabetes educators; HbA1c at baseline, 3, and 6 month post-programs were obtained from a chart review.

Across different studies, most control groups consisted of participants who did not receive any interventions regarding management of DM2. However, they still received standard primary medical care. For example, in Davis et al. (2010), the control group received one 20-minute DM2 education session, using ADA materials, conducted individually at the time of randomization by the nurse in addition to usual care. In Greenwood et al. (2015), the control group—in contrast to the intervention group who used an in-home touchscreen tablet computer connected to a glucometer via USB cables—received regular DM2 education booklets and referral for formal DM2 education as needed. Both groups in Greenwood et al.’s (2015) study continued to receive nurse care coordination, including reminders for health checkups sent through mail; they also discussed behavior changes by telephone and/or secure messaging and discussed possible medication changes with their primary care provider through the online messaging function.

**Summary of the intervention used**. From the methods of delivery used in the review, all the studies utilized telemonitoring either through internet websites, portal messages, video conferences, telephone follow up, or text messages. Four of the ten studies involved telehealth coordination with a nurse or certified diabetes educator and data collection done by research assistants. Two out of ten studies analyzed data directly from an online patient portal to provider or physician. The remaining four studies used only research assistants to facilitate data collection and telemonitoring with participants.

In the four studies which involved nurse telemonitoring, including Davis et al. (2010), Greenwood et al. (2015), Tang et al. (2013), and Welch et al. (2015), participants were able to interact with the nurse through electronic portal messaging or by telephone to discuss the HbA1c data they entered through a web portal or an electronic database. All four of these studies had statistically significant decreases in HbA1c levels. Tang et al. (2013), which was the nurse-led, personalized healthcare program, had the greatest HbA1c decrease at 6 months.

**Measurements and Outcomes**. The primary outcome for all studies was a decrease in HbA1c. Some studies also measured secondary outcomes, such as cholesterol levels, medication adherence, self-efficacy, performance of self-care tasks, quality of life, DM2-specific knowledge (see Table 1). In terms of measurement for HbA1c, examples included HbA1c readings in Arora et al. (2014) which were measured using the Afinion AS-100 Food and Drug Administration-approved capillary point-of-care meter, which reports National Glycohemoglobin Standardization Program-aligned values. Also, in Davis et al. (2010), analysis of HbA1c was performed on an Olympus AU400 via immunoassay/absorption spectroscopy.

In Arora et al. (2014), the primary outcome of median HbA1c decreased by 1.05% in the intervention group compared with 0.60% in the control group (95% confidence interval [CI] 0.27 to 1.17 with a *P*-value of 0.23). Similar trends in the secondary outcomes were noted, such as medication adherence. The intervention group had improvement of nearly 1.0 (out of 8 points), compared to a net decrease of −0.1 in the control group (95% CI 0.1 to 2.1, *P* = 0.025). In terms of satisfaction, the intervention group rated higher in the TExT-MED program than the control group, and they would recommend it to a family or friend with DM2.

In the study by Capozza et al. (2015), both the intervention and control group’s average HbA1c decreased from baseline at 90 and 180 days. There was also no statistically significant difference between the intervention and control groups in terms of change in HbA1c (*P* > 0.05). One reason may be due to a quality improvement effort underway in participating clinics, which increased improvement in the control group. There were also no statistically significant relationships between study duration or interaction and HbA1c reduction. Although no significant reduction in HbA1c was noted, the participants in general were satisfied with the intervention. Of the 53% or 30 intervention group participants who completed the patient satisfaction questionnaires, a mean total satisfaction score of 27.7 out of 32 suggests a high level of satisfaction with the Care4Life intervention program.

## Discussion

This systematic review attempted to determine the best ways of using technology to manage DM2 in ethnic minority populations so that the results can be applied in the care of other ethnic populations, such as Chinese Americans. The next section discusses key factors from our systematic review which may help future researchers develop culturally appropriate telemedicine interventions to help Chinese Americans manage their DM2.

### Best Telemedicine Intervention in DM2 Management

Overall, the greatest reduction in HbA1c was the nurse-led, personalized healthcare program by Tang et al. (2013). In this 12-month study, the intervention group had their data analyzed by nurses through wireless monitoring tools, and each participant was provided with advice and was also sent text or video clips. As a result, the intervention group significantly reduced their HbA1c level of −1.32% at 6 months compared to the control group without using technology.

Another study by Davis et al. (2010) also had a significant decrease of −1.1% at 6 months and −1.2% at 12 months, though not as pronounced as Tang et al. (2013). Participants in the study by Davis et al., a 12-month, 13 session interactive-videoconferencing study had DM2 Telecare which involved the use of a nurse for remote comprehensive DM2 self-management education.

Both studies by Tang et al. (2013) and Davis et al. (2010) involved the use of a nurse and direct feedback from their glucose data. However, there was greater minority population in Davis et al. (2010) than Tang et al. (2013), with 75% African American and 41% minority (African American, Asian, Native Hawaiian, American Indian, Hispanic) respectively. Both studies showed that a telemonitoring intervention to reduce HbA1c with the use of a nurse is the best for racially ethnic minorities. Although not mentioned by the aforementioned studies, it is noteworthy that in order to achieve better DM2 management, a culturally and linguistically competent nurse is needed for better disease management for racially ethnic minorities (Li, Wu, & Chen, 2016).

### Length/Duration of Study

The length of the studies varied from 3 months to 3 years with an average length of 1 year. With a majority (90%) of studies ranging 6 to 12 months in length, significant reductions in HbA1c tended to be significant by at least 6 months, indicating that the best intervention length was at least 6 months long. However, there were no definitive answers on how long home monitoring, text messaging, or telephone interventions should be in order to effectively manage DM2. It is important to remember that DM2 control is a life-long condition even though the completed interventions had a short duration of 6 to 12 months. Data beyond one year warrant further investigation.

### Barriers to DM2 Management

**User-unfriendly devices.** One of the most significant barriers to DM2 self-management is the burden of tracking daily BG readings and medication intake to manage the disease. It was noted that certain electronic devices, such as the personal digital assistant (PDA) in the study by Vuong et al. (2012) and the electronic pillbox in the study by Welch et al. (2015) were user friendly, useful, and convenient. With more individuals owning their personal cell phones, reaching patients through user-friendly telemedicine may be a solution to tackling the DM2 burden.

**Expense**. Cost concerns managing DM2 using technology also exist. Capozza et al. (2015) had significant front-end labor required to recruit and enroll patients given that both the format and the technology were new to many participants. Thus, the cost-effectiveness of telemedicine interventions should be further studied to see if its benefits outweigh the costs.

### Limited Recruitment of Asian Americans

Very few studies involving technological management of DM2 recruited Asian populations. Chung et al. (2017), with the most Asian participants at 32%, was only a retrospective analysis of messages from an online patient portal to physician providers. Furthermore, they did not distinguish the different ethnic groups among the Asian participants in the study. Cultural barriers, including language and culturally different views and traditions to manage illnesses, explains a lower participation of ethnic minorities in clinical interventions for DM2 (Jang, Johnson, D’Eramo-Melkus, & Vorderstrasse, 2018).

In terms of recruitment strategies, a majority of studies (75%) did not indicate their methods to recruit racial minorities such as Chinese Americans. A systematic review done by Li, Wu, and Chen (2016) showed that the best strategies to recruit Chinese Americans is through referral from health professionals and gatekeepers (e.g., community leaders), and patients because Chinese Americans usually connect better with someone they know and trust (Li et al., 2016). For future telemedicine studies, using referral from a primary care provider community leaders/gatekeepers and patients as a primary recruitment approach is advised.

### Other Factors

Of the ten studies, five of them produced insignificant decreases in HbA1c. It is important to learn the reasons behind the insignificant findings so future researchers would be able to design a more efficient telemedicine intervention. In the study by Arora et al. (2014), it was determined that the change in HbA1c between control group and the intervention group were insignificant. A contributing factor may be due to the purely unidirectional mobile text message reminder coordinated by non-nurse research assistants to enforce medication adherence. Therefore, it may be better to use a two-way text messaging system to produce for better results. Capozza et al. (2015) also reported results that were statistically insignificant perhaps partly due to not having a nurse coordinator. Another reason for a decrease in efficacy of the intervention group is due to possibly reflecting a broader quality improvement effort to current DM2 management protocol taking effect in participating clinics (e.g., increased a positive effect of usual care in the control group). The simultaneous quality improvement effort might obscure differences in data from control and intervention patients (Capozza et al., 2015). Additionally, Glasgow et al. (2012) did not detect any significant drop in HbA1c between groups possibly due to attrition and selection bias of participants. Participants who were more active in DM2 management may have a higher tendency to participate in the studies regardless of their group assignment. In this case, even participants in the control group may also seek other methods to help control their DM2 which brings down the difference against the intervention group.

It is suggested that further studies investigate the efficacy of user-friendly telemedical devices, such as smartphones, tablets, prick-less glucometers, and smartwatches as well as a culturally and linguistically competent nurse in helping Chinese Americans manage their DM2 to obtain optimal DM2 management outcomes (i.e., reduction in HbA1c) for short-term (less than 6 months), intermediate (6–12 months) and longterm (longer than a year). It is also important to recruit participants through various clinical sites to generalize the results to a greater population of Chinese Americans.

## Conclusion

Health disparities among racial ethnic patients with DM2 have been and will continue to be a concern in the United States (Chow et al., 2012). With the risk for DM2 at a lower BMI for Asian Americans, including Chinese Americans, this disparity undoubtedly contributes to this seemingly never-ending economic burden. Simultaneously, management of DM2 is evolving due to advances in technology. Given this, we conducted a systematic review with a purpose to answer the question we proposed, “How can we incorporate the use of technology for Chinese Americans to effectively manage their DM2?” More specifically, we anticipated to review literature to discover what could be an effective technology-based intervention which assists and complements limited time for in-person PCP visits to augment the personal aspect of patient-physician interaction (Chung et al., 2017). Several studies in this review outlined their effectiveness in reducing HbA1c levels through use of telehealth and having a nurse facilitate care within 6 to 12 months. With today’s available technology, DM2 patients can be more involved in their own care. At its core, telemonitoring will facilitate active patient participation to manage this chronic disease, and it is of utmost importance to manage as complications (e.g., heart attack) can easily arise if left untreated. In sum, the aforementioned technology-based DM2 management approaches have potential to be adopted and applied to Chinese Americans.

It is worthy to note that although the population used within this review had some data (30% average) about racial ethnic minorities, recruitment of Chinese Americans was sparse. Investigation into recruiting more Chinese Americans is needed to make the results be more meaningful for this population. In addition, our review showed that the use of telehealth through the help of a culturally and linguistically competent nurse may be the solution to minimizing this current burden of DM2 care for Chinese Americans in the long run.

## Declaration of Conflicting Interests

The authors declared no potential conflicts of interest concerning the research, authorship, or publication of this article.
